# Dysbiosis of Oral and Gut Microbiomes in SARS-CoV-2 Infected Patients in Bangladesh: Elucidating the Role of Opportunistic Gut Microbes

**DOI:** 10.3389/fmed.2022.821777

**Published:** 2022-02-14

**Authors:** S. M. Rafiqul Islam, Md. Javed Foysal, M. Nazmul Hoque, H. M. Hamidullah Mehedi, Md. Abdur Rob, Asma Salauddin, Afsana Yeasmin Tanzina, Sabuj Biswas, Sajjad Hossain Noyon, A. M. A. M. Zonaed Siddiki, Alfred Tay, Adnan Mannan

**Affiliations:** ^1^Department of Genetic Engineering and Biotechnology, Faculty of Biological Sciences, University of Chittagong, Chattogram, Bangladesh; ^2^School of Molecular and Life Sciences, Curtin University, Bentley, WA, Australia; ^3^Department of Gynecology, Obstetrics and Reproductive Health, Bangabandhu Sheikh Mujibur Rahman Agricultural University, Gazipur, Bangladesh; ^4^Department of Medicine, 250 Bedded General Hospital, Chattogram, Bangladesh; ^5^Department of Pathology and Parasitology, Chattogram Veterinary and Animal Sciences University, Chattogram, Bangladesh; ^6^Helicobacter Research Laboratory, Marshall Centre for Infectious Disease Research and Training, School of Biomedical Sciences, University of Western Australia, Perth, WA, Australia

**Keywords:** COVID-19, SARS-CoV-2, Bangladesh, metagenomics, oral and gut microbiome

## Abstract

Coronavirus disease-2019 (COVID-19) is an infectious disease caused by SARS-CoV-2 virus. The microbes inhabiting the oral cavity and gut might play crucial roles in maintaining a favorable gut environment, and their relationship with SARS-CoV-2 infection susceptibility and severity is yet to be fully explored. This study investigates the diversity and species richness of gut and oral microbiota of patients with COVID-19, and their possible implications toward the severity of the patient's illness and clinical outcomes. Seventy-four (*n* = 74) clinical samples (gut and oral) were collected from 22 hospitalized patients with COVID-19 with various clinical conditions and 15 apparently healthy people (served as controls). This amplicon-based metagenomic sequencing study yielded 1,866,306 paired-end reads that were mapped to 21 phyla and 231 classified genera of bacteria. Alpha and beta diversity analyses revealed a distinct dysbiosis of the gut and oral microbial communities in patients with COVID-19, compared to healthy controls. We report that SARS-CoV-2 infection significantly reduced richness and evenness in the gut and oral microbiomes despite showing higher unique operational taxonomic units in the gut. The gut samples of the patients with COVID-19 included 46 opportunistic bacterial genera. *Escherichia, Shigella*, and *Bacteroides* were detected as the signature genera in the gut of patients with COVID-19 with diarrhea, whereas a relatively higher abundance of *Streptococcus* was found in patients with COVID-19 having breathing difficulties and sore throat (BDST). The patients with COVID-19 had a significantly lower abundance of *Prevotella* in the oral cavity, compared to healthy controls and patients with COVID-19 without diabetes, respectively. The altered metabolic pathways, including a reduction in biosynthesis capabilities of the gut and oral microbial consortia after SARS-CoV-2 infection, were also observed. The present study may, therefore, shed light on interactions of SARS-CoV-2 with resilient oral and gut microbes which might contribute toward developing microbiome-based diagnostics and therapeutics for this deadly pandemic disease.

## Introduction

Coronavirus disease-2019 (COVID-19) is a contagious disease of the respiratory system, caused by a highly pathogenic and virulent virus known now as SARS-CoV-2. COVID-19 was declared a pandemic after its rapid global spread and the disease to date has accounted for about 4.7 million deaths and more than 232 million confirmed cases (1). SARS-CoV-2 is a zoonotic virus and its reservoir, as studies suggest, is bats. Hence, COVID-19 can be considered as a case of zoonosis. The virus spreads in humans through aerosols, direct contact, respiratory droplets, and fecal-oral contaminations (2). Although COVID-19 is a respiratory disease, it has the potential to affect the human microbiome in both infected and uninfected individuals imposing severe health complications. The inhaled SARS-CoV-2 virus particle binds to the human angiotensin-converting enzyme 2 (hACE2) receptor on epithelial cells lining the respiratory and digestive tracts of the patients, starts replicating, migrates down the respiratory tract along the conducting airways, and a more robust innate immune response is triggered (3, 4). It is assumed that during this propagation, migration, and immune response, the microbiomes throughout the respiratory airways and digestive systems might be altered or changed, and some of them may contribute to further complicating the disease progression (5, 6). The most common clinical features of COVID-19 include fever, dry cough, fatigue, sore throat, diarrhea, difficulty in breathing or shortness of breath, chest pain or pressure, and loss of speech or movement (5–7). The coinfection of the SARS-CoV-2 with other microorganisms is a very important factor in COVID-19 pathogenesis that may complicate the accurate diagnosis, treatment, prognosis of COVID-19, and even increase the mortality rates. Recent evidence from clinical trials and metagenomic investigations have indicated the coexistence of other viruses, bacteria, archaea, and fungi with SARS-CoV-2 in patients with COVID-19 (5, 6, 8). A number of reports have shown that nearly half of the patients who died of COVID-19 apparently had secondary bacterial infections, which further intensified the pathophysiological progression of COVID-19 (9, 10). So far, several risk factors have been identified to be linked with the severity of COVID-19 problems, including genetics, comorbidities, age, and gender (11, 12).

Dysbiosis is defined as a disruption to the microbiome homeostasis caused by an imbalance in the microflora, changes in their functional composition and metabolic activities, or a shift in their local distribution (13). The human microbiome is crucial for the development and maintenance of immunological homeostasis, and it is well-recognized that microbiota imbalance or dysbiosis is strongly linked to a variety of disorders (6, 14). The intestinal tract and oral cavity, which contain the largest and second-largest microbiotas in the human body, respectively, play key roles in the development of infectious diseases (15). The microbiomes of the oral-gut axis have been shown to influence the outcome of numerous infectious diseases by manipulating the host's mucosal immunity in previous investigations (16, 17). Recently, Balmant et al. reported that the microbial populations in the gut microbiome have been associated with COVID-19 disease severity (18). As bacterial coinfections and secondary bacterial infections are noticeable in patients infected with SARS-CoV-2, studies suggest that oral antibiotics can reduce the mortality of patients with COVID-19. Microbiome dysbiosis apparently influences SARS-CoV-2 progression and the severity level in the clinical condition of a patient (18).

The SARS-CoV-2 infection course is critical for dysbiosis in the ecology and dynamics of the human gut microbiome, in both the short term and long term, which in turn influences the human host's health (19, 20). Previous studies suggest that hospitalized patients with COVID-19 exhibit a significant reduction in gut microbiome diversity with depletion of beneficial bacterial symbionts and enrichment of opportunistic pathogens, including *Actinomyces, Rothia*, and *Streptococcus* (20, 21). Another research group showed that decreased gut microbiome diversity could be useful as a COVID-19 severity indicator (13).

Dysbiosis caused by an imbalance in the microflora in the oral cavity has been linked to many other systemic inflammatory or infectious diseases (13). Previous studies on the microbiome of patients with COVID-19 are based on the gut microbiome in general where the oral microbiome has not been explored yet. The present study was focused on analyzing the gut–lung axis based on the oral and gut microbiome of patients with COVID-19. Given the emerging association between the human microbiomes (oral-gut) and SARS-CoV-2, and the unknown driver for patients with COVID-19 suffering from long-lasting symptoms, this study aimed to explore if oral and gut microbiome dysbiosis are associated with clinical symptoms of patients with COVID-19. It is hypothesized that the oral and gut microbiomes are involved in the development of COVID-19, and could serve as a potential diagnostic tool. To test this hypothesis, 16S rRNA MiSeq sequencing using the stool and saliva specimens from 22 patients with COVID-19 (with 15 healthy people as control) were employed to analyze gut and oral microbiome dysbiosis and also related genomic functional perturbations. Characterizing dysbiosis in the context of COVID-19 will allow the assessment of the magnitude of gut flora impairment and relate it with the clinical complications that are observed in different individuals, which often vary from one individual to another. Analyzing microbiome and their dysbiosis can also give an overview of the dominant microbial communities in an individual's microbiome and whether these are pathogenic or beneficial. Individuals can be suggested with personalized therapeutics to restore their microbiome for preventing complicated diseases in the future.

## Materials and Methods

### Ethical Approval

The protocol was approved by the Institutional Review Board (IRB#00981) of 250 Bedded General Hospital, Chattogram, Bangladesh.

### Design of the Study

A cross-sectional study was performed to characterize the oral and gut microbiome and local response in patients with COVID-19 compared to healthy subjects. All participants were recruited from the COVID-19 isolation unit, 250 Bedded General Hospital, Chattogram. Study participants were recruited between June 2020 and September 2020. Verbal and written consent was taken from all the study participants.

### Subject Recruitment and Sample Collection

All study subjects were recruited from the 250 Bedded General Hospital, Chattogram. For this study, patients with COVID-19 (*n* = 22) and healthy people (*n* = 15) >18 years old were selected. From the subjects, stool and saliva samples were collected for further analysis. Pregnant women, breastfeeding mothers, and patients not willing to participate were excluded from the research work. Demographic data, symptoms, history of comorbidities, food habits, medication history, biochemical reports, contact history, hygiene practice, and COVID-19 clinical history were collected from the study population. After two consecutive positive real-time RT-PCR results between 7 and 10 days of symptoms onset, patients with suspected SARS-CoV-2 infection were confirmed as patients with COVID-19 and were recruited as study participants. Within 8 weeks of infection, 50% of the patients were given medication and 50% were not, whereas healthy controls were given no medication before their recruitment in this study.

### DNA Extraction and 16S rRNA Sequencing

Genomic DNA from 74 specimens (gut *n* = 44 and oral *n* = 30) of patients with COVID-19 and healthy controls were extracted using the microbiome DNA purification kit (Thermo Fisher Scientific, USA) following the manufacturer's instructions. The concentration and purity of DNA samples were checked by NanoDrop spectrophotometer 2000c (Thermo Fisher Scientific, USA), Qubit 3.0 fluorometer (Invitrogen, Life Sciences, USA), and agarose gel (1%, w/v) electrophoresis. The extracted DNA was amplified by targeting the V3–V4 regions of the 16S rRNA gene with 341F (5′-CCTACGGGNGGCWGCAG-3′) and 806R (5′-GACTACHVGGGTATCTAATCC-3′) primers (22). The PCR amplification was performed in 50 μl of a final volume containing 25 μl 2′ Hot Start Taq Master Mix (2′; New England BioLabs Inc., USA), 2 μl of DNA, 1 μl (0.2 μM) of each primer, and 21 μl of nuclease-free water. Thirty-five cycles of amplification were performed in an EP Gradient Thermal Cycler (Eppendorf, Germany) according to the conditions recommended for Hot Start Taq Mix with an annealing temperature of 50°C for 40 s. Beads clean-up of PCR products, amplicon barcoding, and pair-end (2 × 300 bp, 600 cycles, v3 chemistry) sequencing of the amplicons were performed under Illumina MiSeq platform (San Diego, CA, USA) according to Illumina standard protocol for 16S metagenomic sequencing.

### Bioinformatics Analysis

The generated paired-end raw reads were assessed for initial quality checking using the FastQC tool (23). The sequence reads were filtered through BBDuk (24) with the following parameters: qtrim = r; trimq = 20; ktrim = r; k = 23; mink = 11; hdist = 1; minlen = 200; tpe; tbo to remove Illumina adapters, known Illumina artifacts; and phiX. Merging of overlapping paired-end reads was performed using NGmerge with default parameters (25). The filtering of chimeric sequences and picking of operational taxonomic units (OTUs) at 0.99 sequence identity threshold was performed using MICCA pipeline (v1.7.0) (26). High-quality reads resulting from this pipeline were further analyzed under two different approaches: taxonomic classification and functional classification. Phylogenetic assignment of each OTU at different taxa levels was performed using the Bayesian LCA-based classification method with a 1e-100 cut-off e-value and 100 bootstrap replications, against NCBI 16S microbial database (27, 28). Multiple sequence alignment (MSA) was performed using clustal omega (ClustalO) (29). Each sample was set to an even depth of 8,465 bp for the analysis of alpha-beta diversity and microbial composition in quantitative insights into microbial ecology (QIIME) software (v1.9.1) (30) and R programming language (v4.1.1) (31). Alpha diversity (observed species richness, Shannon, and Simpson-estimated) was performed in “microbiomeSeq” (https://github.com/umerijaz/microbiomeSeq) and “phyloseq” (32) R packages. Beta-diversity (weighted and unweighted UniFrac distance metrices) was measured through “phyloseq,” “microbiome,” and “ape” (33) R packages. In addition, the metagenomic function of 16S rRNA data set in healthy control and COVID-19 community was predicted following PICRUSt2 pipeline (https://github.com/picrust/picrust2) (34) in support of KEGG database.

### Statistical Analysis

The statistical analysis of beta-ordination was calculated as permutational analysis of variance (PERMANOVA) in the vegan (35) R package. The rarefied data was converted into log1p fold-changes to identify statistically significant bacteria at the genus level in different groups. Genera with Kruskal–Wallis *p* < 0.05 were subjected to Wilcoxon rank-text (unpaired) for multiple comparisons with Bonferroni correction to avoid false discovery rate (FDR) from rarefied data (36). Linear discriminant analysis (LDA) with an LDA cut-off value of 2.0 and more was used to find differentially expressed pathways (37). A *p* ≤ 0.05 was considered statistically significant in all stages of data analysis.

## Results

### Sequence Statistics

**Figure 1:**
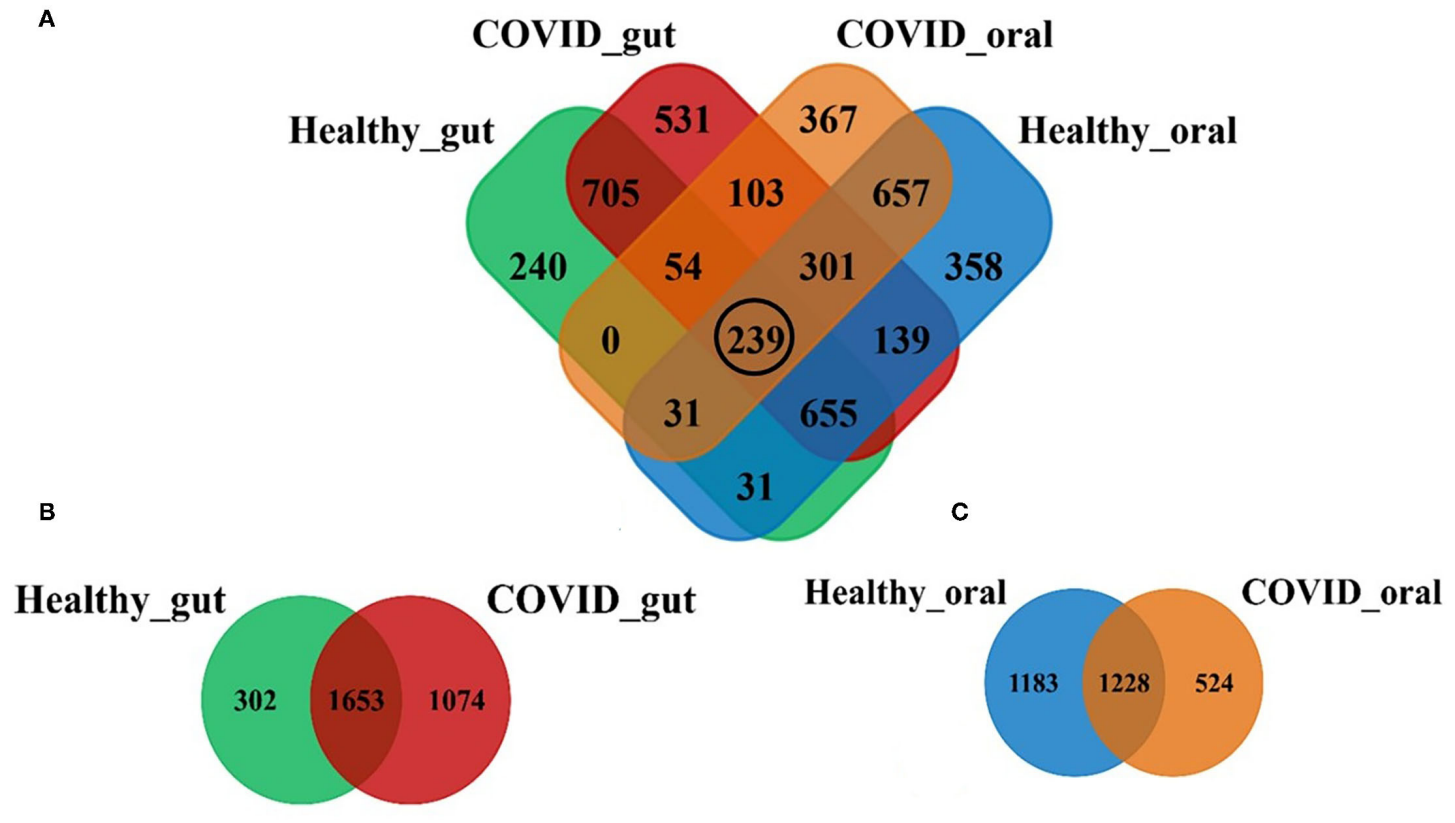
Distribution of shared and unique operational taxonomic units (OTUs) in the gut and oral wash of healthy controls and COVID-19 cases. **(A)** Venn diagram showing the overview of common and unique OTUs among four groups of samples. The black circle indicates the number of OTUs shared to all groups. **(B,C)** Represents the distribution of OTUs between healthy and COVID-gut, and healthy and COVID-oral samples, respectively.

Illumina MiSeq sequencing yielded 1,866,306 paired-end reads from 74 samples, with an average of 26,286.2 ± 978.8 reads per sample. The rarefaction depth curve (Supplementary Figure 1) and good's coverage index (0.998 ± 0.001) indicated that each sample was sequenced at an adequate saturation level to capture maximum bacterial diversity at different taxa levels. After filtering and trimming of low-quality reads, and adapter removal, a total of 1,785,506 reads were retained. Subsequently, 96.8% of the reads (1,728,198) were merged, generating 4,411 OTUs, 21 phyla, 156 families, and 231 classified genera. The sequences from the gut samples (*n* = 44) of both COVID-19 and healthy controls were mapped to 3,029 OTUs while oral samples (*n* = 30) were aligned to 2,935 OTUs. Figure 1 shows the distribution of shared and unique OTUs in the gut and oral wash of patients with COVID-19 and healthy controls. Remarkably, out of these OTUs, only 239 (5.42%) were found to be shared among the gut and oral samples of patients with COVID-19 and healthy controls (Figure 1A). By comparing the distribution of these OTUs according to sample groups, we found that the gut samples of patients with COVID-19 and healthy controls possessed 2,727 and 1,955 OTUs, respectively, of which 54.57% OTUs were shared between the conditions (Figure 1B). Moreover, among these gut-associated OTUs, 1,074 (35.46%) had a sole association with SARS-CoV-2 infections (COVID-19 cases). On the other hand, oral samples from patients with COVID-19 and healthy humans possessed 1,752 and 2,411 OTUs, respectively, with 41.84% OTUs sharing between the two groups. The oral samples from patients with COVID-19, however, had a sole association of only 524 (17.85%) OTUs (Figure 1C).

### Microbial Diversity

**Figure 2:**
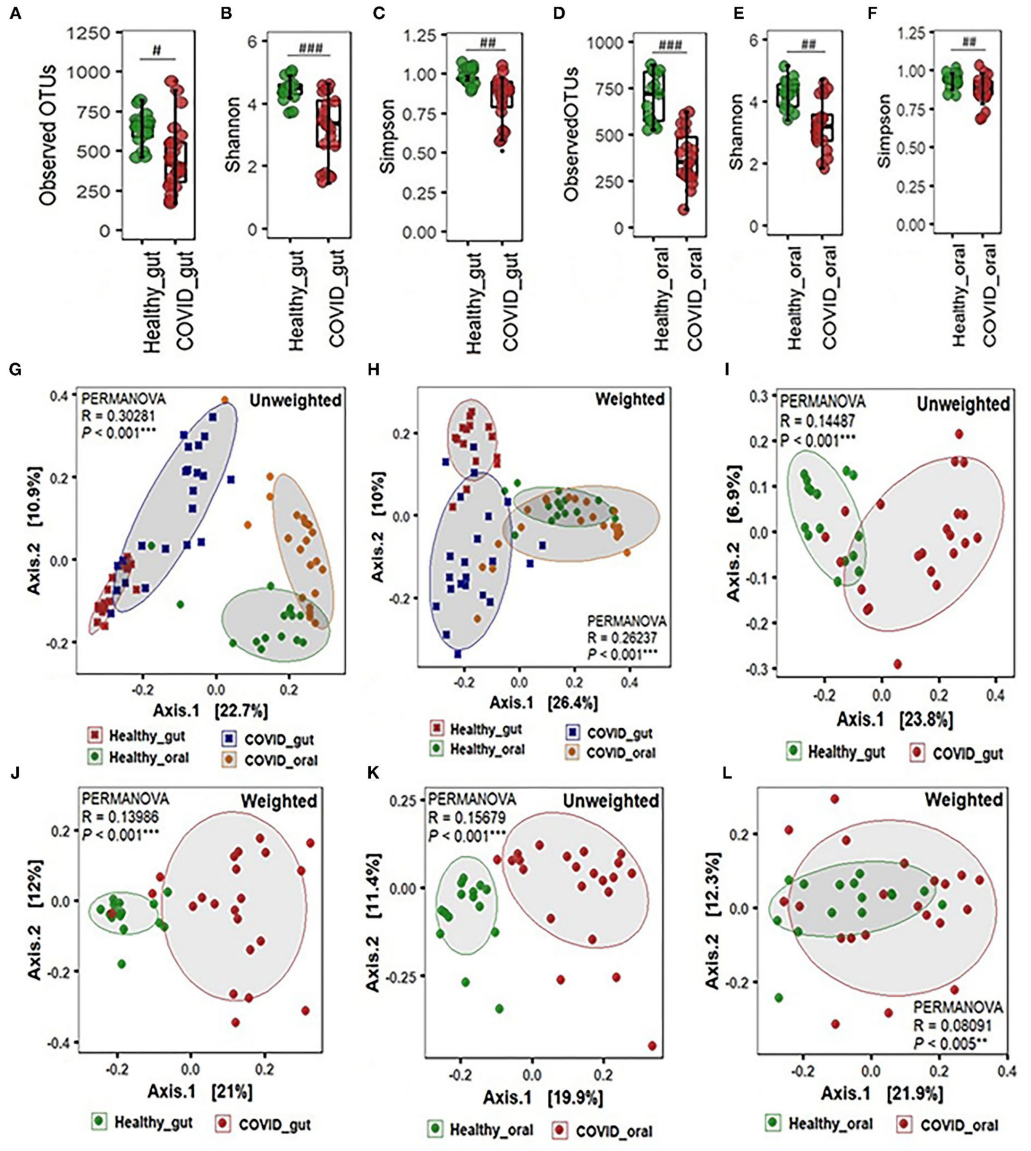
Alpha-beta diversity of microbiota in the gut and oral wash of healthy controls and COVID-19 cases. **(A–F)** α-Diversity measurements of the gut and oral microbiota in terms of observed operational taxonomic units, Shannon, and Simpson index, respectively. **(G)** Unweighted (presence-absence) and **(H)** weighted (relative abundance) UniFrac metric for the gut and oral microbiota of healthy control and COVID-19 cases. **(I)** Unweighted and **(J)** weighted UniFrac metric for the gut microbiota of healthy control and COVID-19 cases. **(K)** Unweighted and **(L)** weighted UniFrac metric for the oral microbiota of healthy control and COVID-19 cases. ^*#*^Significantly different at alpha-level of 0.05. ^*##*^Significantly different at alpha-level of 0.005. ^*###*^Significantly different at alpha-level of 0.001. ^**^Significantly different at alpha-level of 0.005. ^***^Significantly different at alpha-level of 0.001.

The alpha-beta diversity of microbiota in the gut and oral wash of COVID-19 cases and healthy controls are illustrated in Figure 2. Amplicon sequencing revealed higher microbial richness in the gut samples of patients with COVID-19 (Figure 2A) and oral samples of healthy controls (Figure 2D). However, no significant difference (*P* = 0.12, Kruskal-Wallis test) was observed in the evenness index between patients with COVID-19 and healthy individuals' gut and oral samples (Figures 2A–F). Infection with SARS-COV-2 significantly decreased diversity in terms of observed OTUs, along with Shannon and Simpson evenness in the gut (Figures 2A–C). Interestingly, similar to gut environments, the SARS-CoV-2 infection significantly reduced microbial diversity in the oral samples. However, the reduction of diversity was more prominent in oral microbiomes compared to gut samples (Figures 2D–F). Beta diversity analyses based on Unifrac phylogenetic-based distances showed differences in the structure of microbial communities between patients with COVID-19 and healthy controls gut and oral specimens (Figures 2G–L). Differences were observed for both unweighted and weighted Unifrac distances (PERMANOVA *p* < 0.005 for both distances) revealing that patients with COVID-19 and healthy controls differ in quality (i.e., presence/absence) and abundance of phylotypes. The principal coordinate analysis (PCoA) plots representing β-diversity showed significant differences (*p* ≤ 0.005, Kruskal–Wallis test) in bacteriome composition in the gut and oral samples of patients with COVID-19 and healthy controls. The variations were more evident in unweighted UniFrac based on the presence or absence of low-abundant and rare taxa in the community. SARS-CoV-2 significantly modulates the gut microbiota signified by the distinct clustering of bacterial OTUs as observed in both unweighted and weighted UniFrac distance metrics (Figures 2G,H). The SARS-CoV-2 infection triggers the inhibition of common and rare bacteria in the oral community as displayed by the qualitative presence-absence unweighted UniFrac distance metric (Figure 2K). The quantitative differences in microbial community structure (weighted), however, were found less significant than unweighted UniFrac (Figure 2L).

### COVID-19 Associated Shifts in the Gut and Oral Microbiomes

**Figure 3:**
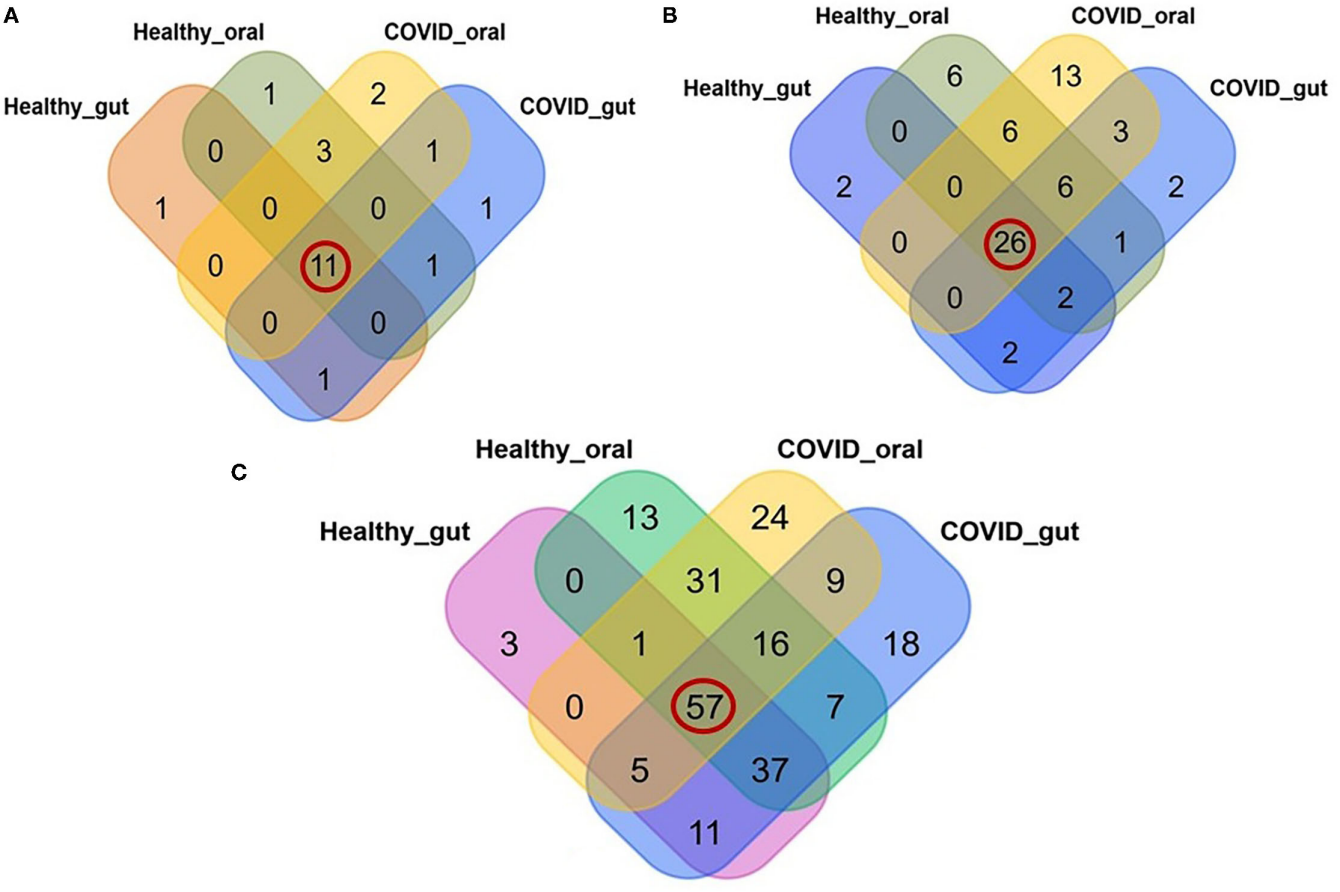
Relative abundance of bacteria at phylum, order and genus level in the gut and oral wash of healthy controls and COVID-19 cases. **(A)** Bacterial abundance at the phylum level. Of 22 detected phyla, 11 (50.0%; highlighted in red circle) were shared across all the sample categories. **(B)** Bacterial abundance at the order level. A total of 69 orders of bacteria were detected, of which 26 (37.68%) were found to be shared across all samples. **(C)** Bacterial abundance at the genus level. A total of 231 bacterial genera were identified in the gut and oral samples of patients with COVID-19 and healthy controls, among which 57 (24.68%) were common to all four groups of samples.

A comparative analysis between patients with COVID-19 and healthy control samples was carried out. Differences in relative abundances of phyla between patients with COVID-19 and healthy control samples were characterized using a Kruskal Wallis non-parametric test. A total of 22 bacterial phyla were detected in the gut and oral samples of patients with COVID-19 and healthy controls, and among these phyla, 50.0% were shared across the sample categories (Figure 3A, Supplementary Material 1). In addition, we successfully detected 69 orders of bacteria in the present study including 42, 54, 32, and 47 in COVID-19 gut, COVID-19 oral, healthy gut, and healthy oral samples, respectively. Notably among these groups, 26 (37.68%) orders of bacteria were found to be shared (Figure 3B, Supplementary Material 1). The oral samples of patients with COVID-19 had sole association of 13 (18.84%, highest) orders followed by the oral samples of healthy controls (6 orders), patients with COVID-19 gut, and gut samples of healthy controls (2 orders in each). The phylum- and order-levels discrepancy of taxonomic compositions was more evident at the genus-level among the four metagenome groups. In this study, 231 genera of bacteria including 160, 143, 114, and 162 in the COVID-19 gut, COVID-19 oral, healthy gut, and healthy oral samples, respectively, were detected. Among these genera, 57 (24.68%) were found to be shared across the four sample groups, and the patients with COVID-19 gut and oral samples had a sole association of 18 and 24 bacterial genera, respectively (Figure 3C, Supplementary Material 1).

**Figure 4:**
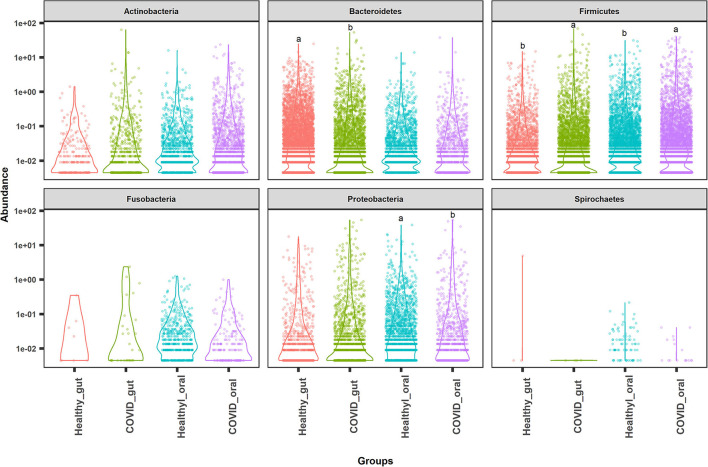
Top abundant bacterial phyla in the gut and oral samples of patients with COVID-19 and healthy controls. *Firmicutes, Bacteroidetes*, and *Proteobacteria* were found to be the most predominant phyla across the sample groups. Each sample group is represented by a different color code. Violin dots with different letters (a▲, b▼) indicate significantly different read abundance at the phylum level between the gut and oral samples with Mann–Whitney *U*-test.

**Figure 5:**
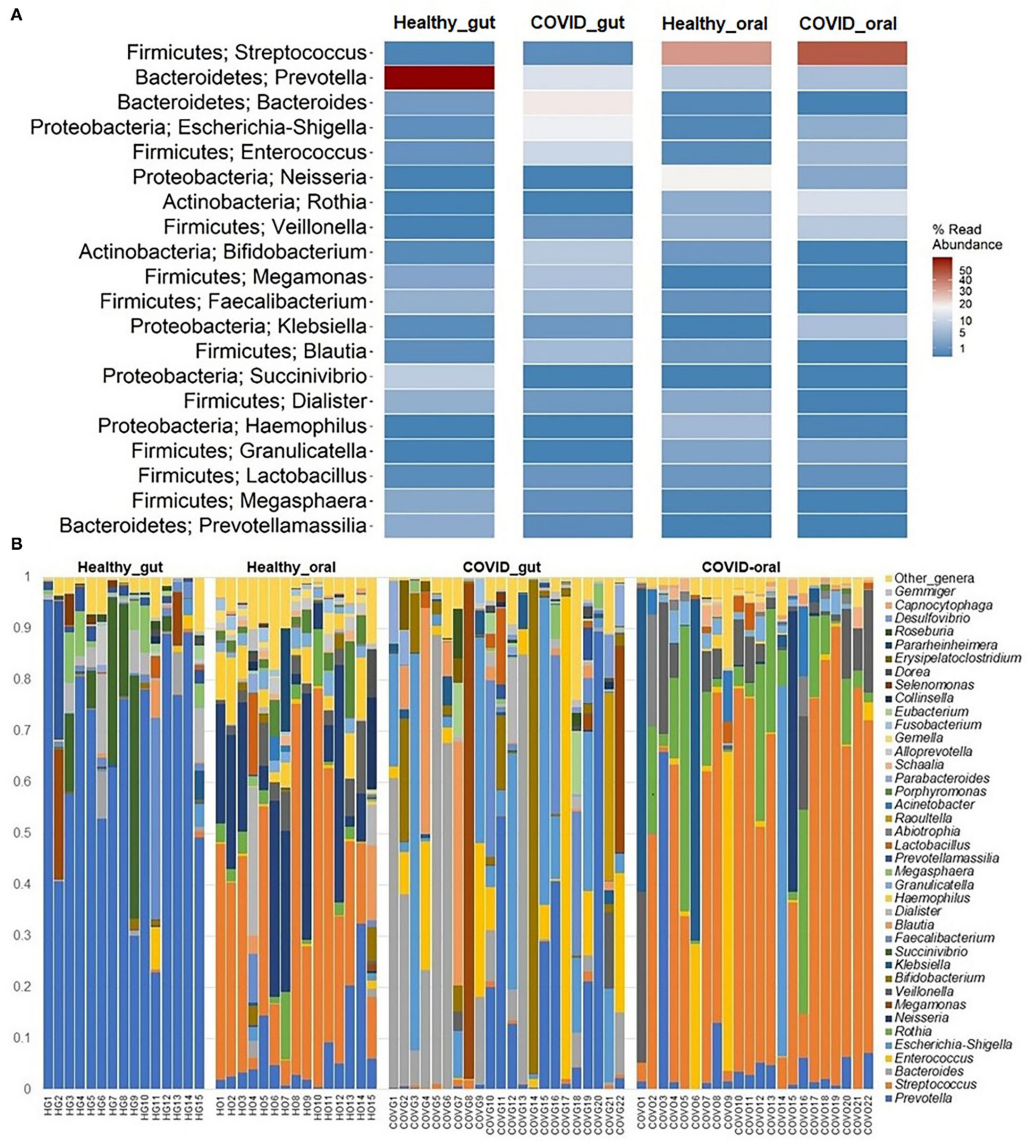
The taxonomic abundance of bacteria in the gut and oral wash of healthy controls and COVID-19 cases. **(A)** Heatmap showing top 20 bacterial genera along with their corresponding phyla in the four sample groups. The color bar represents the abundance value (%) of each genus ranging from 1 (lowest abundance; expressed in dark blue color) to 100 (highest abundance; expressed in brick red color) in the corresponding sample group. **(B)** Relative abundance of 39 bacterial genera in the gut and oral samples of healthy control and patients with COVID-19. The abundance is sorted from bottom to top by the decreasing pattern of bacteria with the remaining genera kept as “other genera.” Each stacked bar represents the abundance of bacterial genera in each sample of the corresponding category.

The most abundant bacterial phyla in the gut and oral samples of patients with COVID-19 and healthy controls are presented in Figure 4. Overall, the gut and oral microbiota of 74 samples were found dominated by *Firmicutes, Bacteroidetes*, and *Proteobacteria* phyla (Figure 4). *Bacteroidetes* (47.2%), *Firmicutes* (28.5%), and *Proteobacteria* (18.9%) phyla comprised 94.6% of the total reads in the gut of patients with COVID-19 and healthy controls (Figures 4, 5A). Oral samples, however, outweighed *Firmicutes* (58.8%), followed by *Proteobacteria* (21.2%) and *Actinobacteria* (10.7%). Unlike in the gut, *Bacteroidetes* (7.7%) richness was found lower in oral samples (Figure 5A, Supplementary Material 1). Predominantly identified bacterial orders in the gut samples of patients with COVID-19 were *Bacteroidales* (32.75%), *Enterobacteriales* (23.37%), *Clostridiales* (13.04%), *Lactobacillales* (11.47%), *Selenomonadales* (9.66%), and *Bifidobacteriales* (7.27%) whereas *Lactobacillales* (54.98%), *Micrococcales* (11.61%), *Enterobacteriales* (10.85%), *Selenomonadales* (6.99%), and *Bacteroidales* (5.33%) were the top abundant bacterial orders in the oral samples of patients with COVID-19 (Supplementary Material 1). Similarly, the gut samples of healthy humans harbored *Bacteroidales* (69.71%), *Selenomonadales* (9.55%), *Aeromonadales* (9.45%), and *Clostridiales* (7.40%) as the predominantly abundant bacterial orders, whereas oral samples of these healthy people possessed *Lactobacillales* (41.02%), *Betaproteobacteriales* (19.36%), *Bacteroidales* (10.41%), *Pasteurellales* (5.44%), and *Selenomonadales* (5.15%) as the top abundant orders of bacteria. The remaining bacterial orders detected in these sample categories had relatively lower (<5.0%) abundances (Supplementary Material 1).

The present microbiome study demonstrated notable differences among the microbial community in patients with COVID-19 and healthy humans at the genus level. Among all the detected bacterial genera (*n* = 231), *Prevotella* (36.7%), and *Streptococcus* (43.3%) were predominant in the gut and oral samples, respectively. *Prevotella* (gut; 36.7%, oral; 5.6%), *Enterococcus* (gut; 6.9%, oral; 4.2%), and *Escherichia, Shigella* (gut; 8.4%, oral; 1.1%) were the only three bacteria that had more than 1% read abundance in both the gut and oral samples (Figure 5A). By comparing the taxonomic abundances of these bacterial genera across the sample categories, we found that *Bacteroides* (19.56%), *Prevotella* (15.74%), *Escherichia* and *Shigella* (14.01%), *Enterococcus* (11.31%), *Bifidobacterium* (8.33%), and *Megamonas* (7.90%) in the COVID-19 gut, and *Streptococcus* (46.06%), *Rothia* (12.11%), *Klebsiella* (7.35%), *Veillonella* (7.22%), *Enterococcus* (6.45%), and *Prevotella* (5.32%) in COVID-19 oral were the predominantly abundant genera (Figure 5B, Supplementary Material 1). Conversely, *Prevotella* (66.31%) and *Succinivibrio* (9.76%) in the healthy gut, *Streptococcus* (38.71%), *Neisseria* (19.36%), *Prevotella* (6.04%), and *Haemophilus* (5.30%) in the healthy oral samples were the top abundant bacterial genera.

The rest of the bacterial genera detected across these four sample groups had relatively lower abundances (<5.0%) (Figure 5B, Supplementary Material 1). The predominant genera in the gut and oral samples of patients with COVID-19 and healthy controls in response to various clinical conditions are documented in Tables 1, 2, respectively. Therefore, SARS-CoV-2 infections significantly (*p* < 0.003, Kruskal–Wallis test) increased the abundance of *Escherichia, Shigella, Enterococcus, Bacteroides*, and *Bifidobacterium* in the gut samples, whereas the richness of *Prevotella* was found to be decreasing (Supplementary Table 1). Among these genera, *Enterococcus* had a higher abundance in patients with COVID-19 who were suffering from loss of motion (LoM) before SARS-CoV-2 infection, compared to patients with motion and healthy controls (Table 1). *Escherichia* and *Shigella* were identified as a signature bacterium in patients with COVID-19 having diarrhea, and medication had a minor effect on these Gram-negative copathogens of SARS-CoV-2. While SARS-CoV-2 infection significantly decreased *Prevotella* growth in the gut of patients with COVID-19, further loss of abundance was observed for patients with medication i.e., general antibiotics including azithromycin, metronidazole, amoxicillin, and ciprofloxacin (Table 1). Unlike the gut samples, the relative abundance of *Prevotella, Neisseria, Haemophilus*, and *Porphyromonas* dropped significantly (*p* < 0.04, Kruskal–Wallis test) in the oral microbiome after SARS-CoV-2 infection (Table 2, Supplementary Table 2). In contrast, among the top abundant bacterial genera, only the relative abundance of *Rothia* increased significantly (*p* < 0.005, Kruskal–Wallis test) after SARS-CoV-2 infections. *Streptococcus* was detected with higher relative abundances in patients with COVID-19 suffering from BDST and LoM (Table 2).

**Table 1:** Major genera in the gut samples of patients with COVID-19 and healthy controls in response to various clinical conditions.

**Loss of taste (LoT)**				
**Bacteria**	**Healthy**	**COVID–LoT**	**COVID + LoT**	**^†^*p*-Value**
*Streptococcus*	1.9 ± 0.4^b^	3.4 ± 0.4^ab^	3.5 ± 0.3^a^	0.01446
*Escherichia-Shigella*	2.6 ± 0.4^b^	7.1 ± 0.6^a^	5.6 ± 0.6^a^	0.00048
*Prevotella*	8.9 ± 0.1^a^	4.4 ± 1.0^b^	5.3 ± 0.6^b^	1.8e-05
*Enterococcus*	1.2 ± 0.6^b^	4.3 ± 0.9^a^	5.1 ± 0.7^a^	0.0003
*Bacteroides*	2.7 ± 0.6^b^	5.5 ± 1.3^ab^	5.6 ± 0.7^a^	0.0152
*Bifidobacterium*	2.9 ± 0.4^b^	5.1 ± 1.0^ab^	4.7 ± 0.5^a^	0.0138
**Loss of motion (LoM)**				
**Bacteria**	**Healthy**	**COVID–LoM**	**COVID** **+** **LoM**	^†^ ***p*** **-Value**
*Streptococcus*	1.9 ± 0.4^b^	3.7 ± 0.4^a^	3.3 ± 0.3^a^	0.01196
*Escherichia-Shigella*	2.6 ± 0.4^b^	6.3 ± 1.0^a^	5.8 ± 0.6^a^	0.00094
*Prevotella*	8.9 ± 0.1^a^	4.1 ± 0.9^b^	5.6 ± 0.6^b^	1.2e-05
*Enterococcus*	1.2 ± 0.6^c^	4.2 ± 0.8^b^	5.2 ± 0.8^a^	0.00027
*Bacteroides*	2.7 ± 0.6^b^	5.1 ± 1.2^ab^	5.8 ± 0.7^a^	0.01394
*Bifidobacterium*	2.9 ± 0.4^b^	5.0 ± 0.8^a^	4.8 ± 0.5^a^	0.01388
**Diarrhoea (DRR)**				
**Bacteria**	**Healthy**	**COVID–DRR**	**COVID** **+** **DRR**	^†^ ***p*** **-Value**
*Streptococcus*	1.9 ± 0.4^b^	3.4 ± 0.3^a^	3.4 ± 0.4^ab^	0.01394
*Escherichia-Shigella*	2.6 ± 0.4^c^	4.8 ± 0.5^b^	8.1 ± 0.2^a^	1.3e-05
*Prevotella*	8.9 ± 0.1^a^	4.9 ± 0.6^b^	5.5 ± 0.9^b^	2.4e-05
*Enterococcus*	1.2 ± 0.6^c^	5.1 ± 0.8^a^	4.5 ± 0.9^b^	0.00038
*Bacteroides*	2.7 ± 0.6^b^	5.5 ± 0.9^ab^	5.6 ± 0.7^a^	0.01498
*Bifidobacterium*	2.9 ± 0.4^b^	5.1 ± 0.6^a^	4.4 ± 0.5^ab^	0.01125
**Medication (MC)**				
**Bacteria**	**Healthy**	**COVID–MC**	**COVID** **+** **MC**	^†^ ***p*** **-Value**
*Streptococcus*	1.9 ± 0.4^b^	3.3 ± 0.4^ab^	3.5 ± 0.3^a^	0.012
*Escherichia-Shigella*	2.6 ± 0.4^b^	5.8 ± 0.8^a^	6.2 ± 0.6^a^	0.00095
*Prevotella*	8.9 ± 0.1^a^	6.2 ± 0.6^b^	4.0 ± 0.7^c^	7.3e-06
*Enterococcus*	1.2 ± 0.6^b^	4.9 ± 0.9^a^	4.9 ± 0.7^a^	0.00041
*Bacteroides*	2.7 ± 0.6^b^	5.3 ± 0.8^ab^	5.8 ± 0.9^a^	0.01388
*Bifidobacterium*	2.9 ± 0.4^b^	4.6 ± 0.6^ab^	5.1 ± 0.6^a^	0.01042

*Genera with at least 1% read abundance in any of the tested groups were considered for the differential abundance analysis. Relative abundance is shown as mean ± SD. Different superscripts (^a, b, c, ab^) placed with mean values in the same column convey their significant difference in bacterial abundance with each other*.

† *p-value adjusted with Bonferroni correction*.

**Table 2:** Major genera in the oral wash of patients with COVID-19 and healthy controls in response to various clinical conditions.

**Loss of taste (LoT)**				
**Bacteria**	**Healthy**	**COVID–LoT**	**COVID + LoT**	**^†^*p*-value**
*Streptococcus*	7.9 ± 0.5^a^	8.1 ± 0.5^a^	7.8 ± 0.3^a^	0.0838
*Prevotella*	6.1 ± 0.3^a^	3.0 ± 1.1^b^	5.1 ± 0.6^a^	0.0405
*Enterococcus*	3.5 ± 0.3^a^	2.7 ± 1.0^a^	4.3 ± 0.5^a^	0.4093
*Rothia*	5.0 ± 0.4^b^	6.8 ± 0.5^a^	6.6 ± 0.4^a^	0.0053
*Veillonella*	5.5 ± 0.4^a^	5.1 ± 0.9^a^	5.6 ± 0.6^a^	0.6152
*Neisseria*	6.7 ± 0.4^a^	2.3 ± 1.4^b^	1.2 ± 0.4^b^	3.1e-05
*Haemophilus*	5.7 ± 0.4^a^	1.5 ± 0.7^b^	1.0 ± 0.4^b^	8.0e-06
*Porphyromonas*	4.8 ± 0.4^a^	0.4 ± 0.3^b^	1.3 ± 0.4^b^	1.5e-05
**Loss of motion (LoM)**				
**Bacteria**	**Healthy**	**COVID–LoM**	**COVID** **+** **LoM**	^†^ ***p*** **-value**
*Streptococcus*	7.9 ± 0.5^a^	8.2 ± 0.3^a^	8.4 ± 0.3^a^	0.1222
*Prevotella*	6.1 ± 0.3^a^	3.6 ± 1.1^a^	5.1 ± 0.6^a^	0.1262
*Enterococcus*	3.5 ± 0.3^a^	3.0 ± 0.8^a^	4.2 ± 0.6^a^	0.6124
*Rothia*	5.0 ± 0.4^b^	6.6 ± 0.5^a^	6.7 ± 0.3^a^	0.0054
*Veillonella*	5.5 ± 0.4^a^	5.3 ± 0.8^a^	5.5 ± 0.6^a^	0.6661
*Neisseria*	6.7 ± 0.4^a^	2.4 ± 1.2^b^	1.1 ± 0.4^b^	2.6e-05
*Haemophilus*	5.7 ± 0.4^a^	2.1 ± 0.9^b^	0.7 ± 0.3^b^	5.0e-06
*Porphyromonas*	4.8 ± 0.4^a^	0.9 ± 0.5^b^	1.1 ± 0.4^b^	2.0e-05
**Diarrhoea (DRR)**				
**Bacteria**	**Healthy**	**COVID–DRR**	**COVID** **+** **DRR**	^†^ ***p*** **-value**
*Streptococcus*	7.9 ± 0.5^a^	8.6 ± 0.5^a^	7.9 ± 0.4^a^	0.0671
*Prevotella*	6.1 ± 0.3^a^	4.4 ± 0.7^a^	5.1 ± 0.9^a^	0.2222
*Enterococcus*	3.5 ± 0.3^a^	4.3 ± 0.4^a^	3.1 ± 1.1^a^	0.3181
*Rothia*	5.0 ± 0.4^b^	6.8 ± 0.3^a^	6.4 ± 0.4^a^	0.00487
*Veillonella*	5.5 ± 0.4^a^	5.0 ± 0.7^a^	5.6 ± 0.6^a^	0.4224
*Neisseria*	6.7 ± 0.4^a^	1.3 ± 0.4^b^	2.0 ± 1.0^b^	3.4e-05
*Haemophilus*	5.7 ± 0.4^a^	1.3 ± 0.5^b^	0.9 ± 0.5^b^	8.5e-06
*Porphyromonas*	4.8 ± 0.4^a^	1.3 ± 0.5^b^	0.4 ± 0.6^b^	1.5e-05
**Medication (MC)**				
**Bacteria**	**Healthy**	**COVID–MC**	**COVID** **+** **MC**	^†^ ***p*** **-value**
*Streptococcus*	7.9 ± 0.5^a^	8.5 ± 0.2^a^	8.2 ± 0.3^a^	0.1576
*Prevotella*	6.1 ± 0.3^a^	4.9 ± 0.7^a^	4.3 ± 0.9^a^	0.1847
*Enterococcus*	3.5 ± 0.3^a^	3.7 ± 0.5^a^	4.0 ± 0.8^a^	0.7931
*Rothia*	5.0 ± 0.4^b^	7.1 ± 0.3^a^	6.2 ± 0.3^ab^	0.0011
*Veillonella*	5.5 ± 0.4^a^	5.3 ± 0.6^a^	5.6 ± 0.8^a^	0.4477
*Neisseria*	6.7 ± 0.4^a^	2.0 ± 0.8^b^	1.0 ± 0.5^b^	2.8e-05
*Haemophilus*	5.7 ± 0.4^a^	1.3 ± 0.4^b^	0.9 ± 0.6^b^	6.8e-06
*Porphyromonas*	4.8 ± 0.4^a^	1.3 ± 0.6^b^	0.8 ± 0.4^b^	1.5e-05
**Breathing difficulty and sore throat (BDST)**				
**Bacteria**	**Healthy**	**COVID–BDST**	**COVID** **+** **BDST**	^†^ ***p*** **-value**
*Streptococcus*	7.9 ± 0.5^b^	7.3 ± 0.2^b^	8.9 ± 0.1^a^	2.7e-05
*Prevotella*	6.1 ± 0.3^a^	4.0 ± 1.1^a^	5.0 ± 0.6^a^	0.1338
*Enterococcus*	3.5 ± 0.3^a^	3.6 ± 1.2^a^	3.9 ± 0.4^a^	0.6134
*Rothia*	5.0 ± 0.4^b^	6.5 ± 0.5^a^	6.8 ± 0.3^a^	0.0039
*Veillonella*	5.5 ± 0.4^a^	4.7 ± 1.1^a^	5.9 ± 0.5^a^	0.4191
*Neisseria*	6.7 ± 0.4^a^	1.4 ± 1.1^b^	1.6 ± 0.5^b^	3.9e-05
*Haemophilus*	5.7 ± 0.4^a^	0.7 ± 0.4^b^	1.4 ± 0.5^b^	7.4e-06
*Porphyromonas*	4.8 ± 0.4^a^	0.3 ± 0.2^b^	1.4 ± 0.5^b^	1.3e-05

*Genera with at least 1% read abundance in any of the tested groups were considered for the differential abundance analysis. Relative abundance is shown as mean ± SD. Different superscripts (^a, b, ab^) placed with mean values in the same column convey their significant difference in bacterial abundance with each other*.

† *p-value adjusted with Bonferroni correction*.

### COVID-19-Associated Changes of Metabolic Features in the Gut and Oral Microbiomes

**Figure 6:**
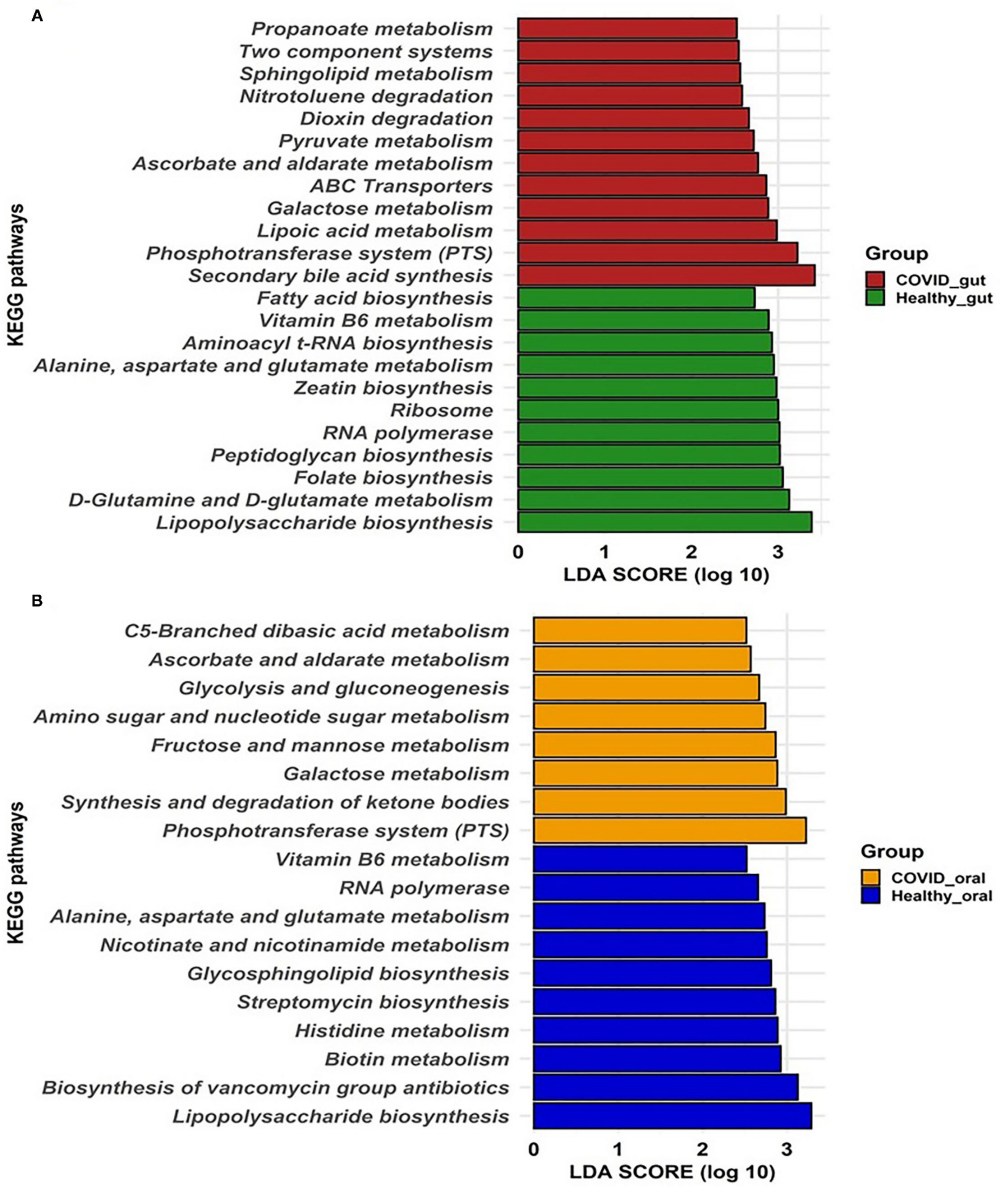
Predicted differentially expressed metabolic pathways in the gut **(A)** and oral wash **(B)** of healthy controls and COVID-19 cases. A total of 23 and 18 KEGG pathways were predicted in the gut and oral samples, respectively. Histogram showing linear discriminant analysis (LDA) score from 0 to 3 represents the expression pattern of pathways in each category of samples.

To gain insight into the relationship between SARS-COV-2 with gut and oral microbiome functions in both patients with COVID-19 and healthy controls, PICRUSt was implemented to predict the potential metagenomes from the community profiles of the normalized 16S rRNA genes. Figure 6 illustrates the predicted differentially expressed metabolic pathways in the gut and oral wash of COVID-19 cases and healthy controls. By comparing the number of genes assigned to each KEGG pathway between the groups, a series of significant differences (*p* = 0.012, Kruskal–Wallis test) were found that lead to the functional divergence in patients with COVID-19- and healthy controls-associated microbiomes. As shown in Figure 6A, 23 significantly different KEGG functional pathways were predicted between the gut microbiomes of patients with COVID-19 and healthy people. Of these KEGG categories, 12 functional categories were highly enriched in the gut microbiomes of patients with COVID-19, including secondary bile acid synthesis, phosphotransferase system (PTS), lipoic acid metabolism, galactose metabolism, ATP-binding cassette (ABC) transporters, ascorbate and aldarate metabolism, pyruvate metabolism, dioxin degradation, nitrotoluene degradation, sphingolipid metabolism, two-component systems, and propanoate metabolism (Figure 6A). In contrast, the gut microbiomes of healthy people were enriched with lipopolysaccharide biosynthesis, D-glutamine and D-glutamate metabolism, folate, peptidoglycan, zeatin and aminoacyl t-RNA biosynthesis, RNA polymerase, ribosome, amino acids (e.g., alanine, aspartate, and glutamate), and vitamin B6 metabolism (Figure 6A). Similarly, the oral microbiomes of patients with COVID-19 had overexpression of gene coding for PTS, synthesis and degradation of ketone bodies, CHO (e.g., galactose, fructose, and mannose) metabolism, amino sugar and nucleotide sugar metabolism, glycolysis and gluconeogenesis, ascorbate and aldarate metabolism, and C5-branched dibasic acid metabolism. Conversely, oral microbiomes of healthy controls showed a higher abundance of metabolic genes related to lipopolysaccharide biosynthesis, biosynthesis of vancomycin groups of antibiotics, biotin, histidine, nicotinate and nicotinamide, amino acids (alanine, aspartate, and glutamate) and vitamin B6 metabolism, and streptomycin biosynthesis (Figure 6B). Therefore, the predicted overexpression of the PTS was observed in both the gut and oral samples from patients with COVID-19, and the expression was highest and second-highest, respectively.

## Discussion

The pathophysiology of SARS-CoV-2 infection is characterized by the concurrent association of diverse microbial consortia that are strongly implicated in the causation of multiorgan dysfunction. Therefore, disease severity in patients with COVID-19 is more likely due to not only viral infection but also from dysbiosis of microbiomes in different systems of the human body (5, 6, 20). Coherently, the oral and gut microbiome of patients with COVID-19 in this study exhibited decreased diversities compared to the healthy controls as reported elsewhere (38). The gut and oral microbiota composition of patients with COVID-19 was found to undergo distinct changes which could modulate different clinical manifestations of COVID-19 including loss of taste, LoM, diarrhea, breathing difficulty, and sore throat. The within (alpha) and between (beta) sample diversities of the microbiomes in the gut and oral samples of both patients with COVID-19 and healthy controls differed significantly (except for evenness indices; *p* = 0.12, Kruskal-Wallis test). The decline in commensal bacterial diversity has been considered a key dysbacteriosis indicator in several diseases (39). This study revealed profound alterations in both oral and gut microbiomes, which were reflected in substantial and significant changes in the community structure identifying potential bacterial marker genera and elucidating the predicted functional profile.

The differences in the dominant microbial phyla (e.g., *Firmicutes, Bacteroidetes*, and *Proteobacteria*) were observed between the two study sampling groups; however, the relative abundance of *Bacteroidetes* remained much lower in oral samples. This trend was also similar between healthy controls and patients with COVID-19 with various clinical conditions. SARS-CoV-2 infection reduced the abundance of *Bacteroidetes*, a known carbohydrate producer (40), which might partially explain the reason for the low functional abundance of carbohydrate transport and metabolism in the COVID-19 groups (41). Patients with COVID-19 had enrichment of pathogenic and commensal bacteria, indicating a degree of microbial changes in clinically ill conditions. This study demonstrated that some orders of bacteria including *Enterobacteriales, Clostridiales, Lactobacillales, Bifidobacteriales*, and *Micrococcales* had higher relative abundances in the gut and oral samples of patients with COVID-19 compared to the healthy controls. The changes in the composition and relative abundances of the microbiomes were more evident at the genus level. These findings indicated that the COVID-19-associated gut and oral samples had a higher relative abundance of bacterial genera such as *Bacteroides, Escherichia* and *Shigella, Enterococcus, Bifidobacterium, Megamonas, Streptococcus, Rothia, Klebsiella*, and *Veillonella*. Differentially abundant levels of *Bacteroides, Prevotella, Escherichia, Shigella, Enterococcus, Bifidobacterium, Megamonas*, and *Actinomyces* were identified in the gut of patients with COVID-19, which is consistent with the findings of several previous studies (38, 42). *Prevotella* species have been overrepresented in patient populations with COVID-19 (43), whereas members of both the *Prevotella* and the *Veillonella* genera have been abundant in the patients with COVID-19 (44).

Members of the *Prevotella* genus have previously been associated with systemic infections, including low-grade systemic inflammation, and are hypothesized to produce proteins that facilitate SARS-CoV-2 infection and increase clinical severity of COVID-19 (43). The increased abundances of these two genera on the tongue have also been associated with an increased risk of death due to pneumonia in older, frail patients (45, 46). Both the *Prevotella and Veillonella* genera are capable of inducing inflammatory responses. *Veillonella* species have shown a strong capacity to induce interleukin-6 (IL-6) (47), whereas *Prevotella* strains primarily activate toll-like receptor-2 (TLR-2) and enhance the expression of inflammatory cytokines, including IL-23 and IL-1 (48). *Rothia* is a species of bacterium found in the mouth that has been linked to T-helper 17 (Th17)-induced lung inflammation and pneumonia in immunocompromised patients (48). The presence of *Rothia* in the gut could indicate that microorganisms migrate from the mouth and respiratory system to the intestines. *Streptococcus* and *Rothia* were associated with secondary bacterial lung infection susceptibility in patients with avian H7N9 virus infection in a previous study (49). Another study has found that Actinomyces-induced changes in the intestinal environment and immunological factors may exacerbate the harm caused by inflammatory bowel disease (50). These alterations could be induced by SARS CoV-2 infection promoting the gut microbiota's protective immunological response, but more investigation is necessary.

Recently, several studies have analyzed the bacterial infection among hospitalized patients with COVID-19 and found a positive correlation between the duration of stay in the hospital and possibilities of acquiring nosocomial infections and coinfections (51, 52). Bacterial genera such as *Prevotella* remained substantially lower in abundance in the gut microbiomes of patients with COVID-19 compared to healthy controls, possibly suggesting a decrease in commensal bacteria and counteraction against SARS-CoV-2 infections (53). Loss of this commensal bacteria (e.g., *Prevotella*) from oral microbiomes indicates that *Prevotella* can act as a local probiotic and counteract with SARS-CoV-2 to defend it in patients with COVID-19 (54). In addition, *Neisseria*, an abundant bacterial genus found in the oral sample of healthy individuals, showed a sharp lower relative abundance in oral samples of patients with COVID-19, signaling the dysbiosis of this genus in the oral microbial community (55). It is worth noticing that a higher relative abundance of *Rothia* in the oral flora of some patients having severe COVID-19 with several comorbidities (such as LoT, LoM, DRR, MC, and BDST) might impact the individual's susceptibility to SARS-CoV-2 through modulating the commensal microbiome composition (38). In a recent study, the genus *Rothia* was found in elevated levels in the gut microbiomes of SARS-CoV-2 and H1N1 influenza patients (53) corroborating our present findings. Loss of pathogenic bacterial genera such as *Haemophilus* and *Porphyromonas* from the oral and gut microbiomes of patients with COVID-19 suggest possible side effects of other cohabiting bacterial communities, such as loss and shortage of necessary dietary nutrients like minerals and micronutrients (56).

The current research also attempted to explore the association of microbiotas with different clinical conditions among patients with COVID-19. In this study, patients with COVID-19 having complications such as loss of taste (LoT) and LoM represented a lower abundance of existing oral bacteria (e.g., *Prevotella, Neisseria, Haemophilus*, and *Porphyromonas*) than patients with COVID-19 without any clinical complications, reflecting possible significant effects of these bacterial genera on the host in maintaining homeostasis. Coherently, patients with COVID-19 with or without difficulty showed similar results i.e., loss of oral *Neisseria* indicating a decrease in oral microbiota diversity. Compared to the gut microbiome, our analysis suggested a stronger impairment in the oral microbiome after SARS-CoV-2 infection, wherein a significant reduction of *Neisseria*, an essential oral microbial genus, was found, along with the suppression of several key metabolic pathways involving the TCA cycle (57). This study also indicated that patients with COVID-19 suffering from diarrhoea possessed higher microbial signature for gastrointestinal pathogens, e.g., *Escherichia and Shigella*, when compared to healthy subjects. Earlier reports also suggested that this gut microbiota contributes to inflammation and disease severity in COVID-19, supporting the present findings (53, 58). Similarly, *Enterococcus* was detected as the signature bacterium in patients with COVID-19 with LoM. Patients with SARS-CoV-2 had a higher abundance of *Enterococcus*, suggesting that respiratory viral pathogenesis seems to be determined also by inflammatory gut microbiomes (53).

Another hallmark of disease severity in COVID-19 is the perturbations of metabolic functions in the gut and oral microbiomes. The KEGG pathways analysis suggested that metabolic pathways differed between SARS-CoV-2 infected patients and healthy individuals. The PTS pathway is essential for ATP production which is used by gut microbiota, particularly in indicating energy-deficient human hosts (59). It was also revealed that genes coding for synthesis and degradation of ketone bodies, CHO metabolism, amino sugar and nucleotide sugar metabolism, glycolysis and gluconeogenesis, ascorbate and aldarate metabolism, and C5-branched dibasic acid metabolism were also found to be overexpressed in the oral microbiomes of patients with COVID-19 compared to healthy subjects. The oral cavity is one of the first entry points in the body and a significant reservoir of SARS-CoV-2; hence, it can be rationally inferred that dysbiosis of the microbiome induced by SARS-CoV-2 infection initially occurs in the oral cavity and subsequently impacts distant microbiomes across the connected body sites *via* the oral–lung or oral–gut axis (6, 60). This finding suggests that the oral microbiome of the patients with COVID-19 may have a lower genetic information processing ability. In particular, membrane transport (e.g., ABC transporters) and cell motility (e.g., bacterial chemotaxis and flagellar assembly) were strongly depleted in the oral microbiomes of the patients with COVID-19.

On the other hand, the lipopolysaccharide biosynthesis pathway was observed to be higher in the gut and the oral site of healthy individuals, reiterating the gut–lung axis relationship (61). Interestingly, bacterial genera such as *Prevotella, Porphyromonas*, produce SCFAs (62), which were severely reduced in abundance (Table 2) in the host due to SARS-CoV-2 infections. The potential association among diverse human microbiota communities, viral particles, and clinical conditions such as diarrhea, loss of movement in human hosts suggest possible microbiota-based treatment along with potential use as an early biomarker for various diseases. The present study, although presented multiple significant outcomes, was not devoid of limitations. First, the number of patients included in the study is low compared to other studies. Second, it was critical to follow up with the patients before and after COVID-19 because of their fear and unwillingness.

The present findings show a clear trend and correlation of microbiota dysbiosis incidence in SARS-CoV-2 infected individuals, substantiating the role of the microbiome as the indicator of disease severity. The microbiome profile of patients with COVID-19 might be impactful in modulating the gut microbiome, introducing healthy microbes inside the body and restoring adverse microbial shifts, and targeted medications. Further in-depth research on microbial functions, host-microbiome interactions during SARS-CoV-2 infection, and investigating the potential of probiotics and prebiotics are recommended to understand the pathogenesis of SARS-CoV-2.

## Conclusion

The human gastrointestinal tract harbors a diverse microbial community that plays important roles in gastrointestinal and respiratory tract diseases, including the regulation of host immunity and preventing the colonization of microbial pathogens. Disrupted homeostasis or dysbiosis indicates a microbial imbalance or maladaptation inside the human body. Collectively, this study reported the alterations in both oral and gut microbiomes of SARS-CoV-2 infected patients with metabolic functional perturbations and made comprehensive analyses to evaluate their potential consequences and implications. The associations between microbial genera with different clinical comorbidities in patients with COVID-19 indicated the potential of microbiome-based intervention in the prevention and treatment of COVID-19. All this information provides new knowledge with innovative perspectives for tackling and managing the ongoing COVID-19 pandemic. The magnitude of the richness of non-commensal microbes can be used as an indicator of the severity of SARS-CoV-2 infection, implying the role of biomarkers. Further attention is required to examine microbial shift and the host-immune responses between patients with COVID-19 and healthy controls in order to explore the actual role of gut microbes in SARS-CoV-2 pathogenesis.

## Data Availability Statement

The original contributions presented in the study are publicly available. This data can be found here: Bioproject PRJNA767939.

## Ethics Statement

The studies involving human participants were reviewed and approved by Institutional Review Board, 250 Bedded General Hospital, Chattogram, Bangladesh. The patients/participants provided their written informed consent to participate in this study.

## Author Contributions

SR, MF, HM, MR, ASi, and AM conceived and designed the experiments. MF, ASa, ATan, SN, SB, ATay, and AM performed the experiments. HM and MR selected and interviewed the patients. MF, MH, ASa, ATan, SB, SN, and AM analyzed the data and drafted the manuscript. SR, MF, MH, ASi, ATay, and AM revised the manuscript. All authors contributed to the article and approved the submitted version.

## Funding

This study was funded by Research and Publication Cell, University of Chittagong (Ref. No: 343/Res/Pub/Cell//CU/2021).

## Conflict of Interest

The authors declare that the research was conducted in the absence of any commercial or financial relationships that could be construed as a potential conflict of interest.

## Publisher's Note

All claims expressed in this article are solely those of the authors and do not necessarily represent those of their affiliated organizations, or those of the publisher, the editors and the reviewers. Any product that may be evaluated in this article, or claim that may be made by its manufacturer, is not guaranteed or endorsed by the publisher.
