# Supplementary material for: Dysbiosis of Oral and Gut Microbiomes in SARS-CoV-2 Infected Patients in Bangladesh: Elucidating the Role of Opportunistic Gut Microbes
Source: Front Med (Lausanne). 2022 Feb 14;9:821777. doi: 10.3389/fmed.2022.821777 (PMC8882723; doi:10.3389/fmed.2022.821777)
Supplement: Supplementary file 1 [file Data_Sheet_1.docx]

**Supplementary file**

**Table S1**. Differentially abundant genera in the gut of COVID-19 patients and healthy controls.

| **Bacteria** | **Healthy_gut** | **COVID-19_gut** | **†*p*-value** |
| --- | --- | --- | --- |
| *Streptococcus* | 1.9±0.4 | 3.4±0.4 | 0.0038 |
| *Escherichia-Shigella* | 2.6±0.4 | 6.0±0.5 | 0.0002 |
| *Prevotella* | 8.9±0.1 | 5.1±0.5 | 0.0000 |
| *Enterococcus* | 1.2±0.6 | 4.9±0.6 | 0.0001 |
| *Bacteroides* | 2.7±0.6 | 5.6±0.6 | 0.0039 |
| *Bifidobacterium* | 2.9±0.4 | 4.8±0.4 | 0.0036 |

*Genera with at least 1% read abundance in any of the tested group were considered for the differential abundance analysis.*

**Table S2**. Differentially abundant genera in the oral wash of healthy control and COVID cases

| **Bacteria** | **Healthy_ oral** | **COVID-19_ oral** | **†*p*-value** |
| --- | --- | --- | --- |
| *Prevotella* | 6.1±0.3 | 4.6±0.6 | 0.0458 |
| *Rothia* | 5.0±0.4 | 6.7±0.3 | 0.0014 |
| *Neisseria* | 6.7±0.5 | 1.5±0.5 | 0.0000 |
| *Haemophilus* | 5.7±0.4 | 1.1±0.4 | 0.0000 |
| *Porphyromonas* | 4.8±0.4 | 1.0±0.3 | 0.0000 |

*Genera with at least 1% read abundance in any of the tested group were considered for the differential abundance analysis.*


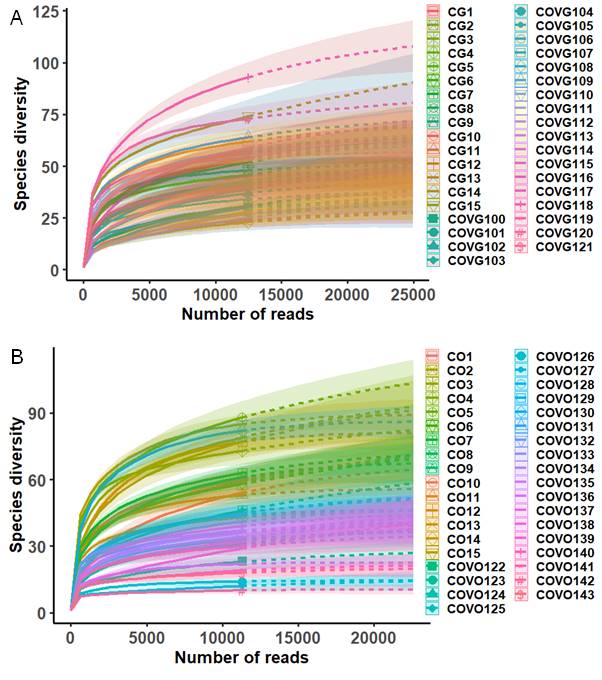


**Figure S1.** Rarefaction curve showing the depth of the sequences in terms of species diversity.
